# Anxiety and depressive disorders in patients with Covid-19

**DOI:** 10.1192/j.eurpsy.2023.742

**Published:** 2023-07-19

**Authors:** A. Stolyarova, N. Tyvina, D. Golovkina, V. Vysokova, J. Zhao, K. Zhang, M. Kinkulkina

**Affiliations:** psychiatry and narcology, Sechenov University, Moscow, Russian Federation

## Abstract

**Introduction:**

Coronavirus infection has shown a significant impact not only on physical health but also on mental health. Among the long-term consequences in the post-COVID period, depressive and anxiety disorders are well observed. A potential mechanism for developing mental disorders after undergoing SARS-CoV-2 is a neuroinflammatory process in the central nervous system.

**Objectives:**

This study aimed to discover the features of anxiety and depressive disorders in people who suffered from COVID-19.

**Methods:**

The study was conducted from October 2021 to September 2022 in outpatient and inpatient conditions of the S. S. Korsakov Psychiatric Clinic of Sechenov University. 58 patients (17 (29.3%) men and 41 (70.7%) women) with a diagnosis of F32, F34.1, F41-F48.0, or F06.3-06.4 according to ICD-10 who underwent COVID-19, mainly of mild and moderate severity (46 (79,3%) and (8 (13,8%) consequently), were examined clinically.

**Results:**

The median age among the respondents was 34 years. Mental illness in the family history had 38 (65.5%) people. Among the examined patients, 21 ((36.2%) people had psychasthenic traits premorbid, 13 (22.4%) – hyperthymic, 9 (15.5%) – hysterical. 15 patients (25.9%) had a history of maternal disorders during pregnancy and childbirth – 16 (27,6%) had neurotic disorders in childhood, 27 (46.6%) had a traumatic brain injury or general anesthesia. 2 patients (3.4%) reported substance abuse, 7 (12.1%) abused alcohol. The median period from somatic well-being after infection to the onset of mental illness was 3 months. The duration from the onset of symptoms of a mental disorder to treatment with a psychiatrist was 3 months. The main symptoms in the clinical picture were impaired concentration (100.0%), decreased productivity (98.3%), sleep disorders (96.6%), decreased mood (98.3%), anxiety (89.7%), anhedonia (75.9%), decreased appetite (65.5%), asthenia (65.5%), emotional lability (44.8%), self-blame ideas (43.1%), obsessive thoughts (39.7%), irritability (37.9%), suicidal thoughts (19.0%).

**Conclusions:**

About two-thirds of respondents were female. The main reasons for consulting a psychiatrist were decreased concentration, sleep disorders, and reduced productivity. In addition, symptoms of anxiety, asthenia, and low mood were revealed, and more than half of the patients complained of apathy. Although the severity of depression was moderate, and the level of anxiety was mild, all these disorders were accompanied by clinically significant fatigue.

**Disclosure of Interest:**

None Declared

